# Supplementary material for: Genetically detoxified pertussis toxin displays near identical structure to its wild-type and exhibits robust immunogenicity
Source: Commun Biol. 2020 Aug 5;3:427. doi: 10.1038/s42003-020-01153-3 (PMC7406505; doi:10.1038/s42003-020-01153-3)
Supplement: Supplementary file 1 — Supplementary Information [file 42003_2020_1153_MOESM1_ESM.pdf]

Supplementary Information

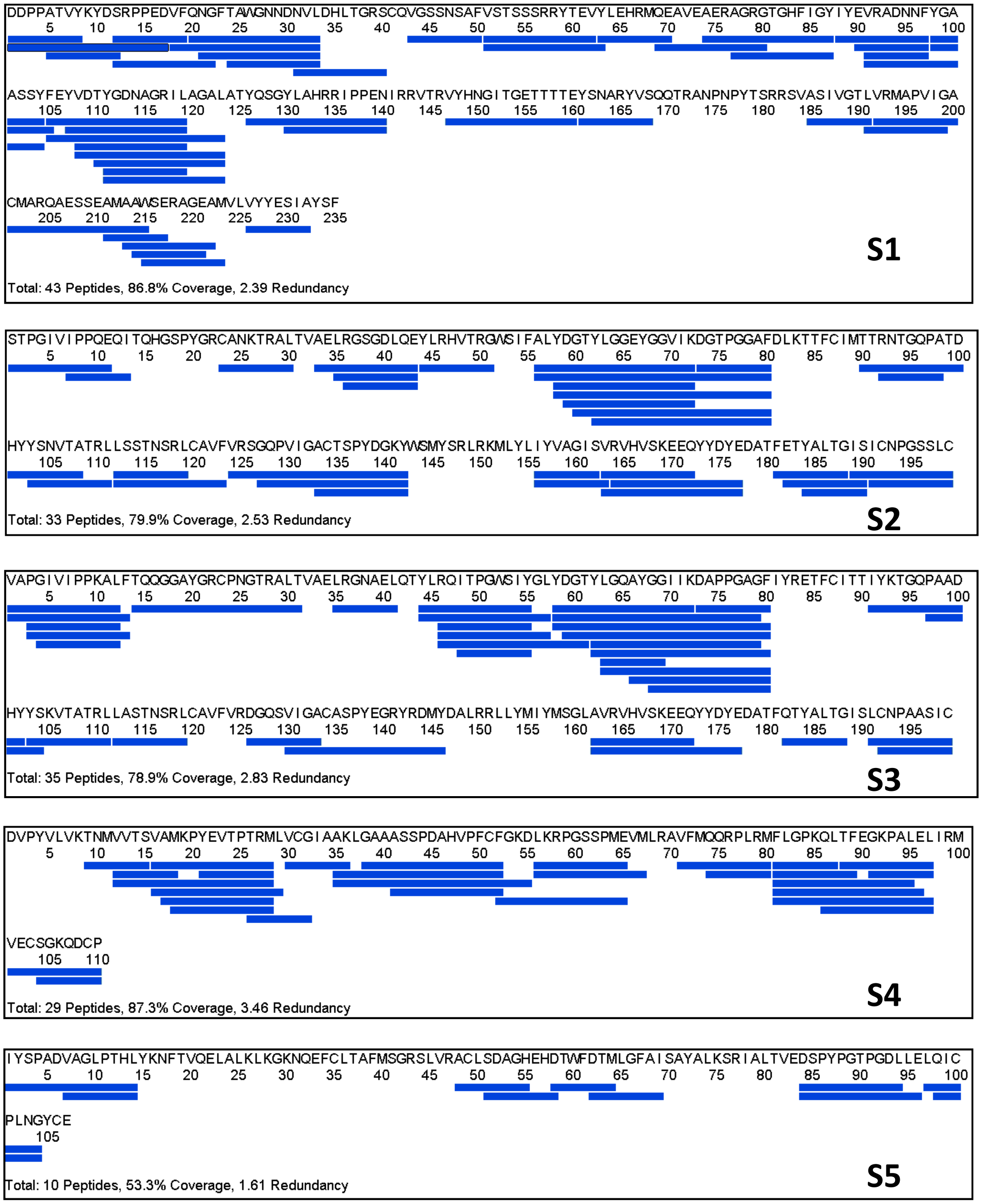

Supplementary Figure 1. Sequence coverage of individual subunits of gdPT.
Digestion of gdPT and PTx yielded an average sequence coverage of 78%. Note that there are two copies of subunit 4 for both gdPT and PTx.

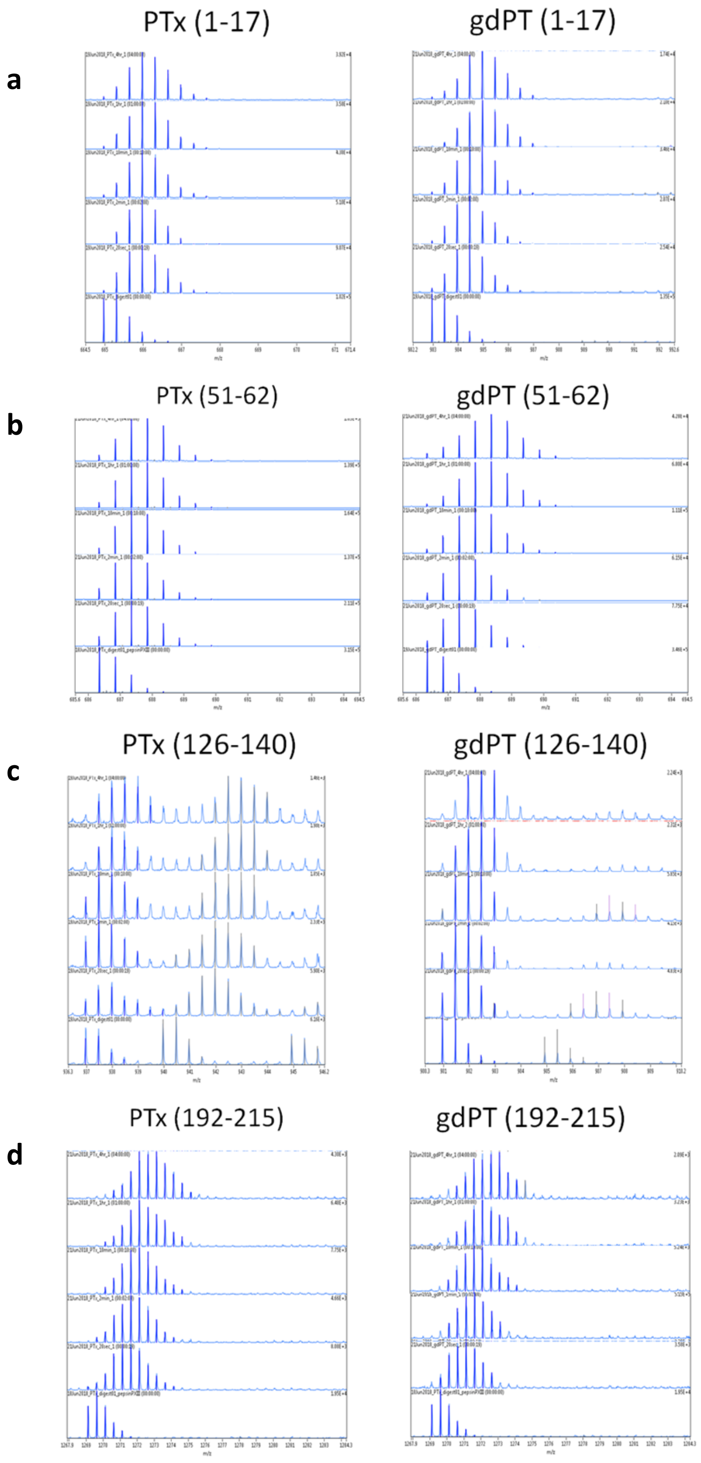

Supplementary Figure 2. Survey mass spectra for HDX-MS time-course experiments showing deuterium uptake. (**a)** Raw mass spectra of peptide residues 1-17 of both PTx and gdPT. This peptide contains mutation R9K for gdPT. **(b).** Raw mass spectra of peptide residues 51-62 of both PTx and gdPT. **(c)** Raw mass spectra of peptide residues 126-140 of both PTx and gdPT. This peptide contains mutation E129G for gdPT. **(d)**Raw mass spectra of peptide residues 192-215 of both PTx and gdPT.

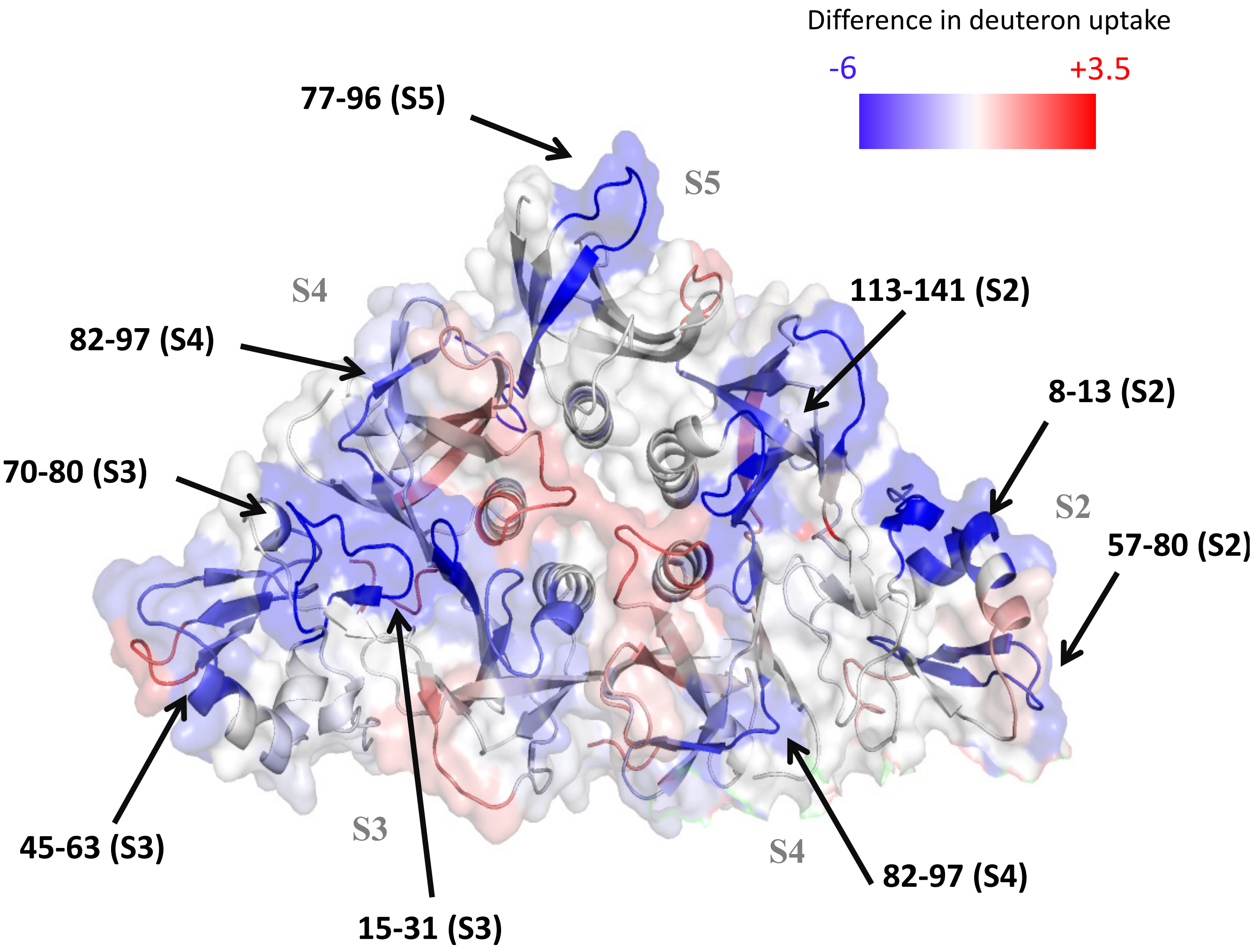

Supplementary Figure 3. Decrease in deuterium uptake in B-oligomer were mainly localized on the interfaces between subunits.
Residues 113-141 of S2 and residues 82-97 of S4, residues 15-31 of S3 and residues 82-97 of S4-1, residues 82-97 of S4-2 and residues 77-96 of S5 showed decrease in deuterium uptake.

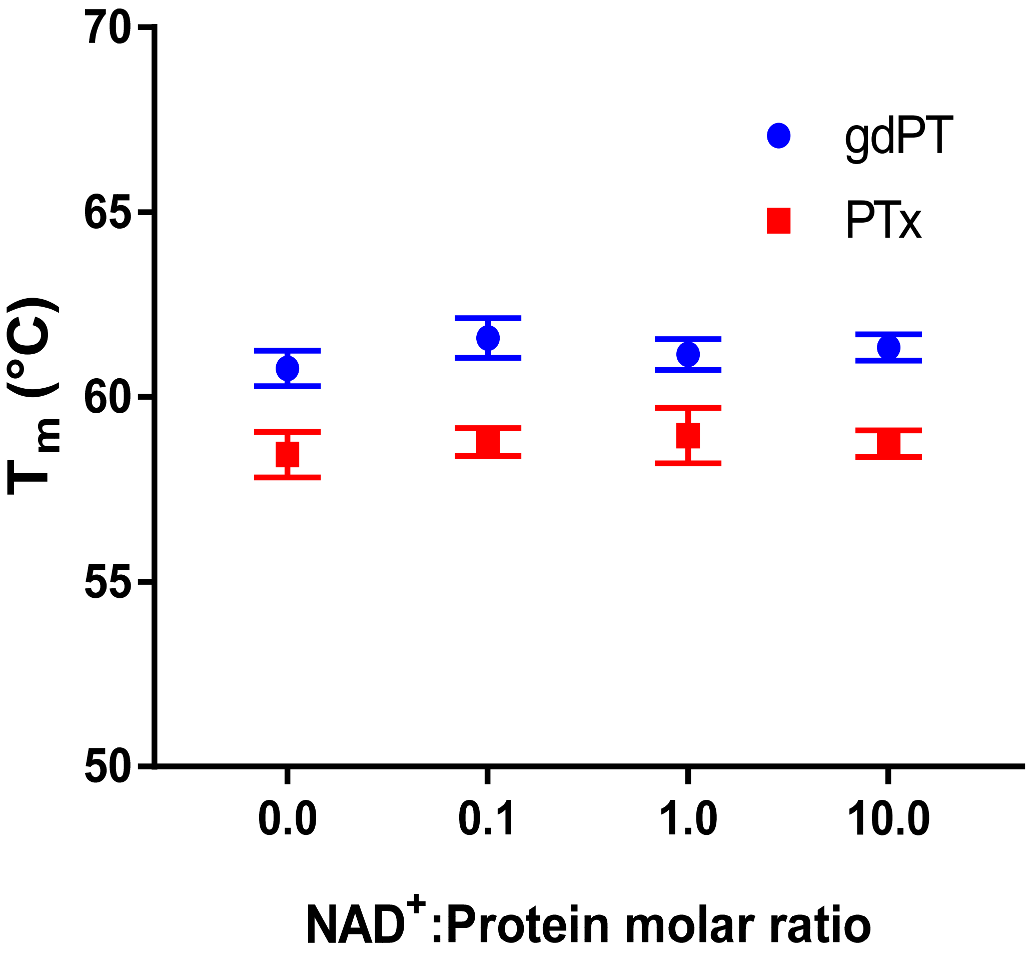

Supplementary Figure 4. Addition of NAD^+ does not impact the thermostability of neither gdPT nor PTx. The effects of NAD^+ on the melting temperature (T_m) of gdPT and PTx when studied by SYPRO orange extrinsic fluorescence.

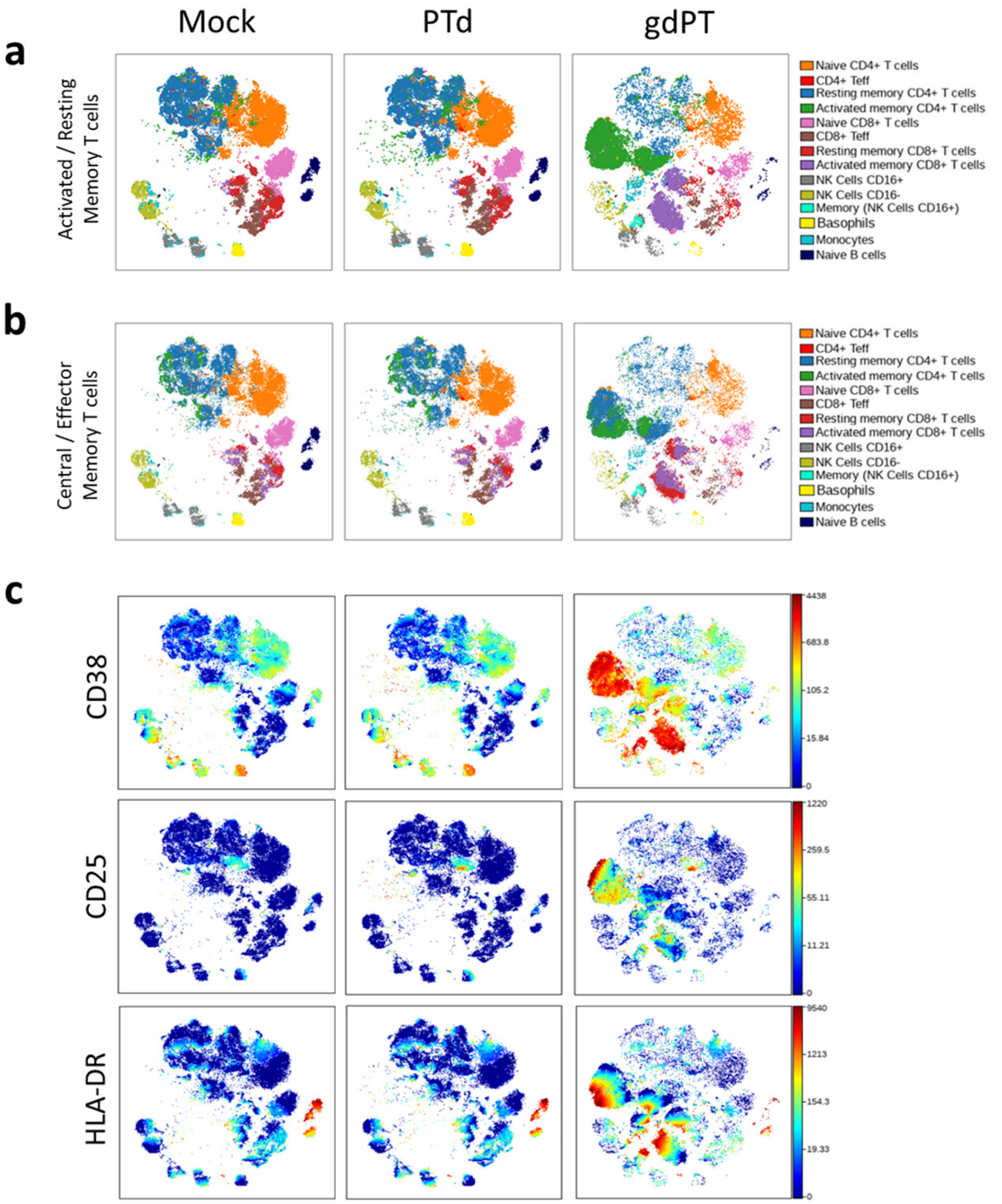

Supplementary Figure 5. viSNE plots and expression marker heatmaps of mock, PTx and gdPT for mass cytometry analysis.

Mass cytometry was used to analyzed whole blood cells cultured for 7 days following stimulation with either Mock, PT or gdPT. A tSNE-based dimension reduction with viSNE was performed on CD45+ CD66b- cells, showing the difference in cell population distribution following whole blood stimulation, each dot represents a single cell. **(a) & (b),** Cell populations defined by manual gating (supplementary Figure 6) projected onto the viSNE plots, each cell population is assigned a specific colour. **(a)** shows memory cells (CD45RO+) gated as either activated (CD38+) or resting (CD38-) memory T cells, whereas **(b)** shows memory T cells gated as central memory (Tcm, CCR7+) and effector memory (Tem, CCR7-). **(c)** Expression of the memory associated marker CD45RO, and the activation markers CD38, CD25 and HLA-DR, presented as a heat map overlay over the viSNE plots. Highlighting activation of memory T cells for both CD4 and CD8 populations.

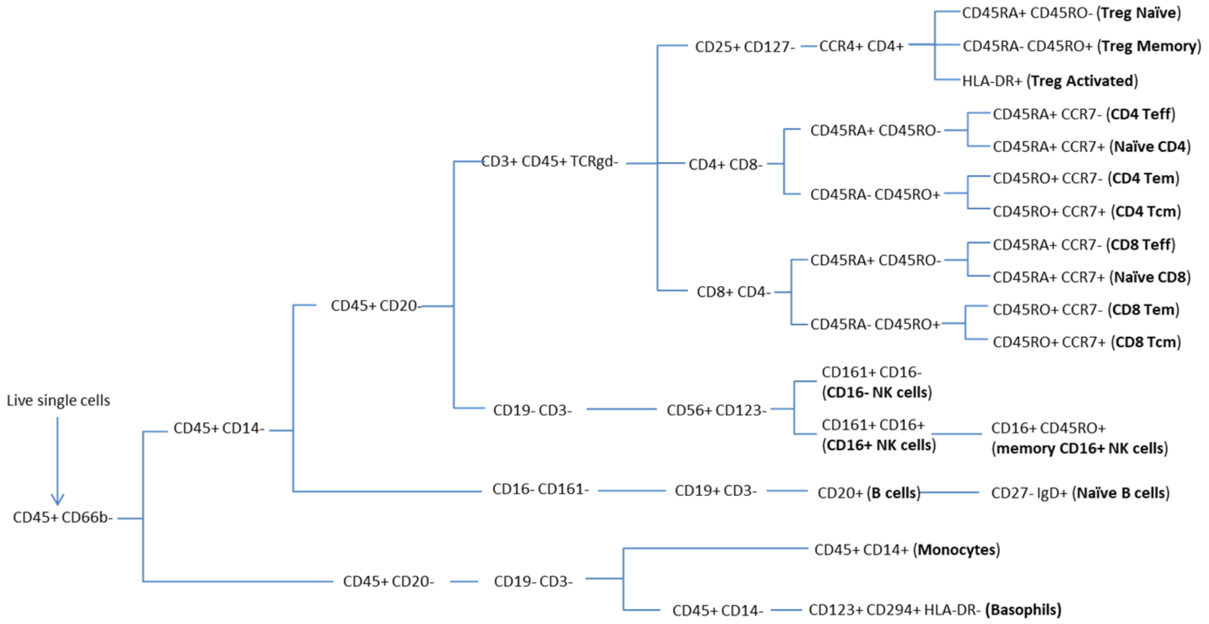

Supplementary Figure 6. Strategy for manual gating of mass cytometry data.
Flow chart describing the strategy used for manual gating of cell population in Cytobank analysis of autologous whole blood

Supplementary Table 1. HDX kinetic rate comparison of gdPT versus PTx for the impacted peptides in subunit 1 result from the double mutation.

Peptide residues	gdPT rates	PTx rates	Ratio of gdPT/PTx rates
1-17*	0.1389	0.0183	7.59
51-62	0.0791	0.0179	4.42
126-140*	0.0505	0.0214	2.36
192-215	4.29, 0.033	5.79, 0.0146	0.74, 2.26

*Contains the mutation sites: R9K and E129G

Supplementary Table 2. Kinetic parameters determined by BLI for calculation of dissociation constants for gdPT and PTx.

[gdPT]	K_{obs}	K_{off}	K_a	K_D
3.00E-06	4.60E-03	3.40E-03	4.00E+02	8.50E-06
1.00E-06	3.90E-03	2.80E-03	1.10E+03	2.55E-06
5.00E-07*	2.60E-03	3.30E-03	-1.40E+03	-2.36E-06

* Outlier, therefore did not use for analysis

K_D (Average)	Std.Dev
5.52E-06	2.98E-06

[PTx]	K_{obs}	K_{off}	K_a	K_D
3.00E-06	1.68E-02	3.70E-03	4.37E+03	8.47E-07
1.00E-06	6.00E-03	3.00E-03	3.00E+03	1.00E-06
5.00E-07	3.80E-03	2.40E-03	2.80E+03	8.57E-07

K_D (Average)	Std.Dev
9.01E-07	6.98E-08

Supplementary Table 3. Percentage of cell population relative to live single cell population. Cell populations were manually gated.

Percentage of cell population (relative to all cells)	Stimulation		
	Mock	PTd	gdPT
Naïve CD4 T cells	18.2	20.5	5.2
CD4 Teff	0.1	0.2	0.1
CD4 T_{CM}	24.6	24.8	39.1
CD4 T_{EM}	3.6	4.3	8.4
Activated CD4 Memory T cells	1.1	1.4	41.6
Resting CD4 Memory T cells	26.5	27.0	4.6
Naïve CD8 T cells	7.7	8.0	1.9
CD8 Teff	5.0	5.1	1.3
CD8 T_{CM}	4.4	4.0	13.7
CD8 T_{EM}	2.7	2.9	6.1
Activated CD8 Memory T cells	0.2	0.3	18.3
Resting CD8 Memory T cells	6.9	6.6	1.4
NK Cells CD16-	3.1	2.8	1.0
NK Cells CD16+	2.7	3.4	1.7
NK Cells CD16+ CD45RO+	0.0	0.0	0.8
Monocytes	0.7	1.0	0.7
Total B cells	3.7	2.9	0.3
Naïve B cells (CD127- IgD-)	2.8	2.2	0.2
Basophils	1.4	2.2	0.2

Supplementary Table 4. HDX back-exchange calculation for 11 enolase peptides

Peptides	RT (min)	D_0	D_{100}	Exch. Backbone	Back Exchange
SVYDSR	3.89	725.76	729.1558	5	32.1
GVLHAVK	3.16	722.89	726.8221	6	34.5
ANIDVK	3.61	658.75	662.3935	5	27.1
NVPLYK	4.24	732.88	735.9442	4	23.4
YDLDFK	5.55	799.88	803.0605	5	36.4
IATAIEK	3.73	744.89	749.2986	6	26.5
LNQLLR	4.49	755.92	760.468	5	9.0
GNPTVEVELTTEK	5.32	1416.55	1424.914	11	24.0
SIVPSGASTGVHEALEMR	5.49	1841.07	1851.667	16	33.8
AVDDFLISLDGTANK	7.01	1578.74	1586.587	14	43.9
TAGIQIVADDLTVTNPK	6.04	1755.99	1765.216	15	38.5
				Average	29.9

Supplementary Table 5. Maxpar Direct Immune Profiling 30-marker Panel used to stain stimulated and cultured whole blood for the mass cytometry assay.

Target	Metal Tag	Clone	Target	Metal Tag	Clone
**Lineage Markers**					
**CD11c**	Sm147	Bu15	**CD3**	Er170	UCHT1
**CD123_IL-3R**	Nd143	6H6	**CD4**	Nd145	RPA-T4
**CD14**	Er168	63D3	**CD45**	Y89	HI30
**CD16**	Nd148	3G8	**CD45RA**	Nd150	HI100
**CD185_CXCR5**	Gd158	J252D4	**CD45RO**	Sm149	UCHL1
**CD19**	Nd144	HIB19	**CD56_NCAM**	Dy163	NCAM16.2
**CD194_CCR4**	Sm152	L291H4	**CD57**	Gd155	HCD57
**CD196_CCR6**	Pr141	G034E3	**CD66b**	Yb172	G10F5
**CD197_CCR7**	Er167	G043H7	**CD8a**	Nd146	RPA-T8
**CD20**	Yb171	2H7	**IgD**	Yb174	IA6-2
**CD294**	Er166	BM16	**TCRgd**	Dy164	B1
**Activation Markers**					
**CD127_IL-7Ra**	Yb176	A019D5	**CD27**	Sm154	O323
**CD161**	Eu151	HP-3G10	**CD28**	Gd160	CD28.2
**CD183_CXCR3**	Gd156	G025H7	**CD38**	Dy161	HB-7
**CD25_IL-2Ra**	Eu153	BC96	**HLA-DR**	Yb173	LN3
