# Supplementary material for: Genetically detoxified pertussis toxin displays near identical structure to its wild-type and exhibits robust immunogenicity
Source: Commun Biol. 2020 Aug 5;3:427. doi: 10.1038/s42003-020-01153-3 (PMC7406505; doi:10.1038/s42003-020-01153-3)
Supplement: Supplementary file 2 — Description of Additional Supplementary Files [file 42003_2020_1153_MOESM2_ESM.pdf]

Description of Additional Supplementary Files

File Name: **Supplementary Data 1**
Description: Summary of all the peptides for both gdPT and PTx used for the HDX analysis

File Name: **Supplementary Data 2**
Description: The source data underlying Figures 4, 5 and 6
