# Supplementary material for: Genetically detoxified pertussis toxin displays near identical structure to its wild-type and exhibits robust immunogenicity
Source: Commun Biol. 2020 Aug 5;3:427. doi: 10.1038/s42003-020-01153-3 (PMC7406505; doi:10.1038/s42003-020-01153-3)
Supplement: Supplementary file 5 — Reporting Summary [file 42003_2020_1153_MOESM5_ESM.pdf]

nature research

Corresponding author(s): D. Andrew James

Last updated by author(s): Jun 15, 2020

Reporting Summary

Nature Research wishes to improve the reproducibility of the work that we publish. This form provides structure for consistency and transparency in reporting. For further information on Nature Research policies, see our Editorial Policies and the Editorial Policy Checklist.

Statistics

For all statistical analyses, confirm that the following items are present in the figure legend, table legend, main text, or Methods section.

n/a	Confirmed	
☐	☒	The exact sample size (n) for each experimental group/condition, given as a discrete number and unit of measurement
☐	☒	A statement on whether measurements were taken from distinct samples or whether the same sample was measured repeatedly
☐	☒	The statistical test(s) used AND whether they are one- or two-sided<br>*Only common tests should be described solely by name; describe more complex techniques in the Methods section.*
☒	☐	A description of all covariates tested
☒	☐	A description of any assumptions or corrections, such as tests of normality and adjustment for multiple comparisons
☐	☒	A full description of the statistical parameters including central tendency (e.g. means) or other basic estimates (e.g. regression coefficient) AND variation (e.g. standard deviation) or associated estimates of uncertainty (e.g. confidence intervals)
☐	☒	For null hypothesis testing, the test statistic (e.g. F, t, r) with confidence intervals, effect sizes, degrees of freedom and P value noted<br>*Give P values as exact values whenever suitable.*
☒	☐	For Bayesian analysis, information on the choice of priors and Markov chain Monte Carlo settings
☒	☐	For hierarchical and complex designs, identification of the appropriate level for tests and full reporting of outcomes
☒	☐	Estimates of effect sizes (e.g. Cohen's d, Pearson's r), indicating how they were calculated

Our web collection on statistics for biologists contains articles on many of the points above.

Software and code

Policy information about availability of computer code

Data collection	MxCube (https://journals.iucr.org/s/issues/2010/05/00/bf5031/)<br>MxPro-Mx3005Pv4.00<br>HDxDirector v 1.0.3.9<br>MassLynx v4.1<br>CyTOF Software [Version 6.5.358]<br>Octet data acquisition Software V 7.0.1.2.6
Data analysis	GraphPad Prisim V7.02<br>autoBUSTERv2.11.7<br>Molrep and Coot(CCP4 software package)<br>autoPROC<br>PyMOL Molecular Graphics System, v 2.0.6 (TM), Schrodinger, LLC<br>DynamX v3.0.0<br>PLGS v3.0.2<br>SigmaPlot v 13.0<br>Cytobank v7.3.0<br>R v3.6.1<br>Octet data analysis software V 7.0.1.5

For manuscripts utilizing custom algorithms or software that are central to the research but not yet described in published literature, software must be made available to editors and reviewers. We strongly encourage code deposition in a community repository (e.g. GitHub). See the Nature Research guidelines for submitting code & software for further information.

Data

Policy information about availability of data

All manuscripts must include a data availability statement. This statement should provide the following information, where applicable:

- Accession codes, unique identifiers, or web links for publicly available datasets
- A list of figures that have associated raw data
- A description of any restrictions on data availability

Atomic coordinates and related structure factors have been deposited in the Protein Data Bank with accession code 6RO0. The source data underlying Figures 4, 5 and 6 are provided as a separate source data file. Any other relevant data are available upon reasonable request.

Field-specific reporting

Please select the one below that is the best fit for your research. If you are not sure, read the appropriate sections before making your selection.

☒ Life sciences ☐ Behavioural & social sciences ☐ Ecological, evolutionary & environmental sciences

For a reference copy of the document with all sections, see nature.com/documents/nr-reporting-summary-flat.pdf

Life sciences study design

All studies must disclose on these points even when the disclosure is negative.

Sample size	Sample sizes were determined based on similar experiments and prior experience from our laboratory. No statistical method was used to predetermine sample sizes
Data exclusions	No data was excluded
Replication	HDX-MS experiments were performed for one biological replicate (protein batch) and three technical replicates. Three replicates were performed for extrinsic fluorescence experiments. The number of replicates for each specific experiment is indicated in the manuscript and figure legends.
Randomization	Randomization is not relevant to the current study
Blinding	Blinding is not relevant to the current study

Reporting for specific materials, systems and methods

We require information from authors about some types of materials, experimental systems and methods used in many studies. Here, indicate whether each material, system or method listed is relevant to your study. If you are not sure if a list item applies to your research, read the appropriate section before selecting a response.

Materials & experimental systems

n/a	Involved in the study	
☐	☒	Antibodies
☒	☐	Eukaryotic cell lines
☒	☐	Palaeontology and archaeology
☒	☐	Animals and other organisms
☐	☒	Human research participants
☒	☐	Clinical data
☒	☐	Dual use research of concern

Methods

n/a	Involved in the study	
☒	☐	ChIP-seq
☒	☐	Flow cytometry
☒	☐	MRI-based neuroimaging

Antibodies

Antibodies used	Antibodies used are included in the Maxpar Direct Immune Profiling Assay kit (Product Cat# 201325)
Validation	Validation report for the antibodies is available on the manufacturer's website.https://jp.fluidigm.com/binaries/content/assets/fluidigm/white-papers/maxpar-direct-immune-profiling-assay_white-paper_.pdf

Human research participants

Policy information about studies involving human research participants

Population characteristics	Human blood used as a reagent in mass cytometry experiment was obtained from healthy donors (male or female) volunteers.
Recruitment	Blood donors volunteered and were evaluated by registered nurses on-site at the Sanofi-Pasteur Occupational Health Center (Toronto, Canada). Donors were screened for infectious diseases and assessed independently by a physician while maintaining full anonymity.
Ethics oversight	The volunteers completed and signed a statement giving informed consent permitting the use of their blood for research purposes.

Note that full information on the approval of the study protocol must also be provided in the manuscript.
